# Bone metastases from breast carcinoma: histopathological – radiological correlations and prognostic features

**DOI:** 10.1038/sj.bjc.6601198

**Published:** 2003-08-12

**Authors:** J J James, A J Evans, S E Pinder, E Gutteridge, K L Cheung, S Chan, J F R Robertson

**Affiliations:** 1 Nottingham City Hospital, Hucknall Road, Nottingham NG5 1PB, UK

**Keywords:** breast carcinoma, bone metastases, prognosis, survival

## Abstract

The aim of this study was to identify factors that may be associated with the development of bone metastases in patients with metastatic breast carcinoma and to see if any of these factors had a bearing on subsequent survival. In total, 492 patients presented to the Nottingham City Hospital with metastatic breast carcinoma between July 1997 and December 2001. Of these, 267 patients had bone metastases at presentation with metastatic disease, 91 patients in this group had bone as their only site of metastatic disease. Sites of first presentation of metastatic disease were prospectively recorded, as were histological features of the primary tumour (tumour type, histological grade, lymph node stage, tumour size and oestrogen receptor (ER) status). The radiological features of the bone metastases, the metastasis-free interval and serological tumour marker levels at presentation with metastases were all recorded. There was a significant association between the development of bone metastases and lower grade tumours (*P*=0.019), ER-positive tumours (*P*<0.0001) and the lymph node stage of the primary tumour (*P*=0.047). A multivariate analysis found that metastasis-free interval, additional sites of metastatic disease other than bone, ER status and serological tumour marker levels all independently contributed to survival from time of presentation with bone metastases.

The survival of patients with metastases is variable ranging from a matter of months to many years. The ability to predict prognosis and response to treatment has a considerable impact on patient management. It is well established that oestrogen receptor (ER) status and site of presentation of metastatic disease have the greatest impact on patient survival, with additional contributions made by patient age, disease-free interval and histological grade.

Bone is the most common site of metastases in patients with breast carcinoma and so patients with bone metastases make up the largest single group of patients presenting with metastatic disease. It has previously been reported that 20% of patients with bone metastases survive for more than 5 years, which emphasises the wide variation in survival seen in this group of patients (Coleman *et al*, 1987). The aim of this study was to identify factors that may be associated with the development of bone metastases and to see if any of these factors had any bearing on subsequent survival.

We have assessed traditional factors such as ER status, histological grade, lymph node stage and size of the primary tumour, patient age, metastasis-free interval and the presence of metastases at sites other than bone. We have examined the radiological appearance of the bone metastases and looked for associations with the histological features of the tumour and patient survival. In common with other centres, we increasingly use serological tumour markers in the diagnosis and monitoring of patients with metastatic breast carcinoma. The prognostic significance of elevated tumour markers at presentation with bone metastases was also studied. Prognostic features were assessed in a univariate and multivariate fashion.

## METHOD

Between July 1997 and December 2001, 492 patients presented to our unit with newly diagnosed metastatic breast carcinoma. The group consisted of patients presenting with metastatic disease following a previous diagnosis of breast carcinoma and patients with metastatic disease at the time of initial diagnosis of breast cancer (stage 4).

Virtually all patients were initially investigated for metastatic disease due to a clinical symptom or sign. At presentation, patients were routinely assessed for metastatic disease in the bones, chest and liver. In the first instance, plain radiographs were used to assess the chest and bones. A radiograph of the pelvis was obtained routinely, supplemented with radiographs of any symptomatic areas. Bone metastases visible on the plain radiograph were recorded as being sclerotic, lytic or mixed. Bone scintigraphy, computed tomography (CT) and magnetic resonance (MR) imaging were used when the diagnosis was uncertain or equivocal. Bone lesions not visible on plain radiographs, but identified on the other imaging modalities were described as occult. The presence of liver metastases was determined using ultrasound or less frequently CT. Sites of metastatic disease at first presentation were prospectively recorded.

Tumour markers were recorded at the time of first presentation. The markers routinely measured at our institution are erythrocyte sedimentation rate (ESR), carcinoembryonic antigen (CEA) and cancer antigen 15.3 (CA15.3). CEA was said to be elevated above a value of 10 ng ml^−1^ and CA15.3 was elevated above a level of 35 U ml^−1^.

Pathological data were obtained from the patients previous mastectomy or wide local excision specimens. Histological grade, lymph node stage, tumour size, ER status and tumour type were recorded for each patient where possible. Tumour types included Ductal No Specific Type (Ductal NST), Tubular Mixed, Lobular, Mixed ductal and lobular and pure tubular; details of the classification of tumour types are well described elsewhere (Ellis *et al*, 1992). The tumour grade was assessed by the Nottingham method (Elston *et al*, 1991). Lymph node stage was determined from the results of axillary node sampling or dissection: stage 1 disease – no nodal involvement; stage 2 disease – three nodes or less containing tumour; stage 3 disease – four or more nodes containing tumour. For patients diagnosed with primary breast cancer before 1995, ER activity was measured using the standard radioligand binding assay on tissue cytosol samples; the threshold for the designation of ER positivity being 10 fmol mg^−1^ of protein. More recently ER status has been determined using immunocytochemical methods, with an H-score >50 identifying ER-positive tumours. For patients presenting with locally advanced primary tumours, ER status was determined by core biopsy at the time of presentation. In this situation, provisional tumour grade obtained from core biopsy was not included. All the pathology was reported by one of three specialist breast pathologists, using standard pathological techniques.

Metastasis-free interval was calculated. This was defined as the interval between first diagnosis of breast cancer and time of first presentation with metastatic disease. Survival from diagnosis of metastases was determined from the hospital computer system of patient attendances and deaths, contacting the patient's general practioner and from the local health authorities patient records.

Throughout the study period patient management remained consistent. Endocrine therapy was the preferred first-line method of treatment in patients with metastatic breast carcinoma. Exceptions to this were patients with widespread or life-threatening visceral metastases. These patients and those with ER-negative tumours were advised to have chemotherapy, if considered fit. The endocrine treatments of choice included the ER antagonist Tamoxifen or an aromatase inhibitor (Anastrozole). Gonadorelin analogues (Goserelin) were used in premenopausal women to achieve ovarian ablation. Bisphosphonates were prescribed routinely for patients with bone metastases according to a defined protocol.

Survival curves were calculated for each factor analysed, using the Kaplan–Meier method. A Cox multivariate analysis was used to establish which of the factors independently contributed towards prolonged survival after the presentation with bone metastases. The statistical analysis was performed using StatView 5.0 for Apple Macintosh (SAS Institute Inc., Cary, NC, USA).

## RESULTS

The distribution of metastatic disease in the patient group is shown in Table 1

**Table 1:** Sites of first presentation of metastatic breast carcinoma

**Site of first presentation of metastases**	**Number of patients (%) (*n*=492)**
*Bone*	267 (54)
Bone metastases only	91
Bone metastases and other metastases	176
Nonbony metastases only	170
Chest	186 (38)
Liver	144 (29)
Ascites	19 (4)
Brain	15 (3)
Ovarian	7 (1)

. Bone metastases were the most frequently observed metastases. In all, 267 (54%) patients had bone metastases as their first site of presentation of metastatic disease. Of these, 170 patients had no evidence of bone metastases at presentation. Baseline bone investigations were not performed or incomplete in 55 patients presenting with metastases at other sites. These patients were excluded from subsequent analysis. In all, 91 patients had bone metastases as their only site of metastatic disease at presentation.

The patient and tumour characteristics of the study population (*n*=437) are shown in Table 2

**Table 2:** Correlation between presentation with bone metastases and primary tumour characteristics

		**Bone metastases at presentation with metastatic disease**		
**Tumour characteristics**		**Yes**	**No**	**Univariate analysis**
Histological grade (%)	1	14 (70)	6 (30)	*χ*^2^=7.90
	2	71 (68)	34 (32)	*P*=0.0193
	3	94 (52)	87 (48)	
ER status (%)	+VE	157 (69)	71 (31)	*χ*^2^=19.755
	−VE	60 (45)	73 (55)	*P*<0.0001
Lymph node stage (%)	1	29 (48)	31 (52)	*χ*^2^=6.13
	2	70 (68)	33 (32)	*P*=0.0467
	3	40 (60)	27 (40)	
Primary tumour size (mm) (%)	<15	23 (55)	19 (45)	*χ*^2^=4.389
	15–30	56 (37)	96 (63)	*P*=0.1114
	>30	35 (40)	52 (60)	
Patient age (%)	⩽40	15 (56)	12 (44)	*χ*^2^=2.889
	41–50	46 (64)	26 (36)	*P*=0.5766
	51–60	56 (56)	44 (44)	
	61–70	59 (60)	40 (40)	
	>70	91 (65)	48 (35)	
Tumour type (%)	Ductal NST	114 (56)	89 (44)	Ductal NST *vs* tubular mixed *χ*^2^=5.03 *P*=0.025
	Tubular mixed	24 (77)	7 (23)	
	Lobular	25 (69)	11 (31)	
	Mixed ductal and lobular	10 (50)	10 (50)	Ductal NST *vs* lobular *χ*^2^=2.22 *P*=0.136
	Pure tubular	2 (67)	1 (33)	

. There is a significant relationship between bone metastases as the first presentation of metastatic disease and histological grade of the primary tumour. Bone metastases were significantly more likely to be associated with a lower grade primary tumour (*χ*^2^=7.90, *P*=0.019). Patients presenting with bone metastases were significantly more likely to have ER-positive primary tumours compared to patients who presented with metastatic disease at other sites (*χ*^2^=19.76, *P*=<0.0001). Bone metastases were also more likely to be associated with lymph node-positive primary tumours (*χ*^2^=6.13, *P*=0.047). Bone metastases were more frequently observed in patients with Tubular Mixed primary tumours compared with Ductal NST (*χ*^2^=5.03, *P*=0.025). No significant association with bone metastases was observed for the other tumour types including lobular carcinomas. Presentation with bone metastases was not related to the patient's age or the size of the primary tumour.

The radiographic appearances of the bone metastases and the tumour characteristics are shown in Table 3

**Table 3:** Association between the radiographic appearance of the bone metastases and the primary tumour characteristics

	**Histological grade (%)**			**ER status (%)**		**Tumour type (%)**				
**Radiographic appearance of bone metastases**	**1**	**2**	**3**	**+VE**	**−VE**	**Ductal NST**	**Tubular mixed**	**Lobular**	**Mixed ductal and lobular**	**Pure tubular**
Occult	3 (21.5)	11 (15.5)	20 (22)	32 (21)	16 (28)	22 (20)	3 (12.5)	7 (28)	0	0
*Visible*										
Lytic	7 (50)	27 (38.5)	38 (41)	59 (39)	18 (31)	48 (43)	9 (37.5)	10 (40)	3 (25)	2 (100)
Mixed	3 (21.5)	14 (20)	16 (17)	30 (20)	11 (19)	19 (17)	7 (29)	3 (12)	4 (33)	0
Sclerotic	1 (7)	18 (26)	18 (20)	31 (20)	13 (22)	22 (20)	5 (21)	5 (20)	5 (42)	0
Univariate analysis	NS			NS		NS				

NS=no significant relationship.

. There was no relationship between whether the bone metastases were visible or occult on plain radiographs and the histological grade of the primary tumour (*χ*^2^=0.10, *P*=0.608) or the ER status of the primary tumour (*χ*^2^=0.98, *P*=0.322). There was also no relationship between the radiographic appearances of the bone metastases (i.e. lytic, sclerotic or mixed) and the grade of the primary tumour (*χ*^2^=2.52, *P*=0.641) or the ER status of the primary tumour (*χ*^2^=0.58, *P*=0.749). There was no relationship between the radiographic appearance of the bone metastases and the type of primary tumour (*χ*^2^=11.81, *P*=0.461).

**Figure 1:**
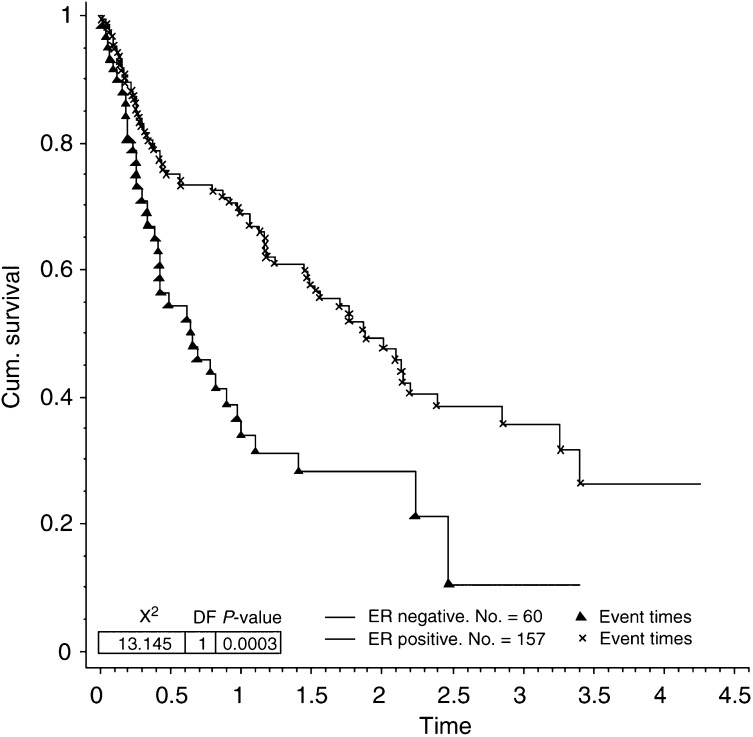
Survival for patients with bone metastases by ER status.

**Figure 2:**
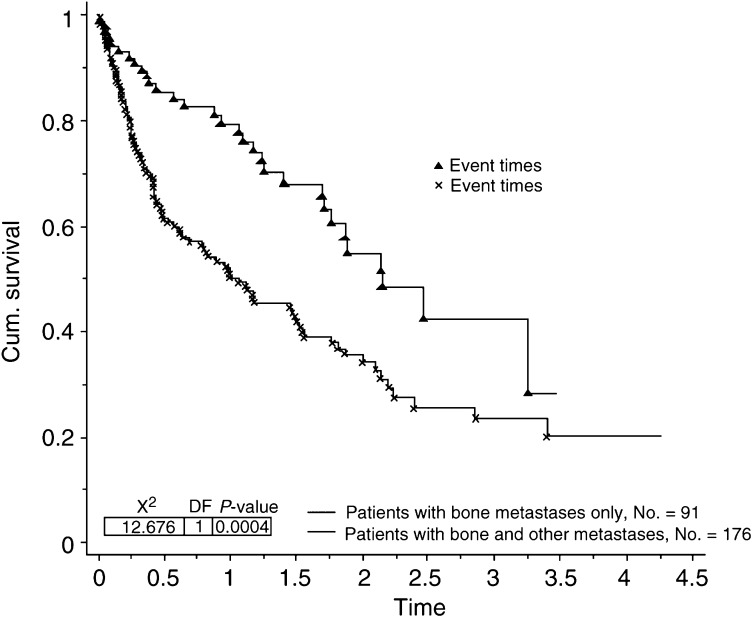
Survival for patients with bone metastases only compared to those with bone and other sites of metastatic disease.

**Figure 3:**
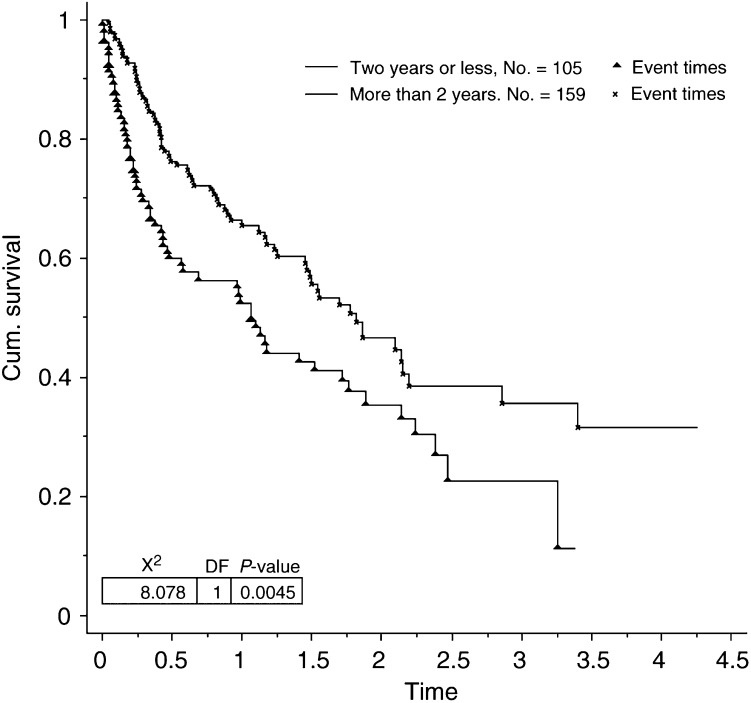
Survival for patients with bone metastases by metastasis-free interval.

**Figure 4:**
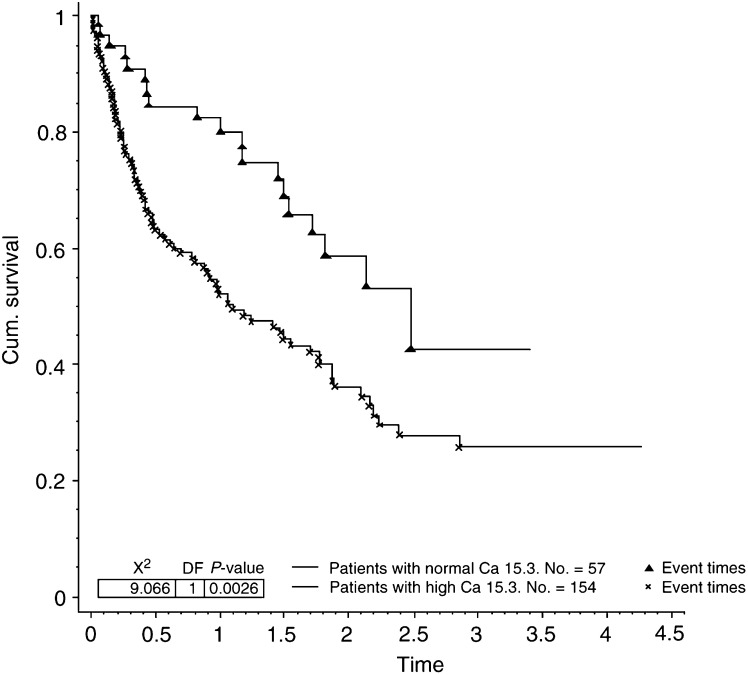
Survival for patients with bone metastases by Ca 15.3 level (cutoff 35).

Seven of the factors analysed were found to contribute significantly towards prolonged survival in patients presenting with bone metastases. These were ER status (*P*=0.0003, Figure 1

), histological grade (*P*=0.034), additional sites of metastatic disease (*P*=0.0004, Figure 2

), patient age (*P*=0.0003), number of hot spots on bone scan (*P*=0.040) and metastasis-free interval (*P*=0.0045, Figure 3

). Levels of the tumour markers CA 15.3 and CEA at presentation were also significantly related to survival (*P*=0.0026 and 0.017, respectively, Figures 4

and 5

**Figure 5:**
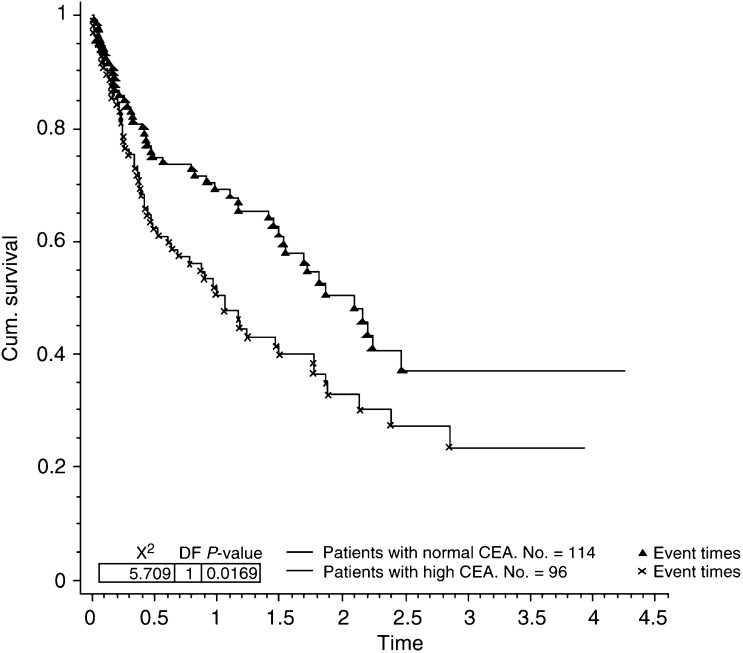
Survival for patients with bone metastases by CEA level (cutoff 10).

). The radiographic appearance of the bone metastases, histological tumour type, lymph node stage, primary tumour size and ESR at presentation with bone metastases were not significantly related to survival.

Three of the factors analysed were also found to contribute significantly to survival in the 91 patients with bone as their only site of metastatic disease. These were ER status (*P*=0.018), Ca 15.3 (*P*=0.025) and metastasis-free interval (*P*=0.004). In the same group of patients there was a trend for worsening survival if the CEA level was elevated at presentation, but this did not reach statistical significance.

A Cox multivariate analysis found that a long metastasis-free interval, absence of metastases at sites other than bone, ER-positive tumours, normal CEA and CA15.3 levels at presentation all contributed independently towards prolonged survival from time of presentation with bone metastases (Table 4

**Table 4:** Cox multivariate analysis

**Factor analysed**	***P*-value**	**Coefficient**
Presentation Ca 15.3	0.0105	0.0003
Metastasis-free interval	0.0411	0.127
ER status	0.0399	0.732
Presentation CEA	0.0055	0.0004
Presence of metastases at sites other than bone	0.0314	0.780

).

## DISCUSSION

Prognostic factors for metastatic breast carcinoma have received less attention than prognostic factors for primary disease. This may in part be due to the inherent difficulty in separating whether a factor is a ‘pure' prognostic factor, a predictive factor for response to therapy or both. For primary breast carcinoma there are historical series where patients received no systemic adjuvant therapy and later series where they did. Consequently, the potential value of factors as prognostic or predictive can be teased out more easily. For patients with metastatic disease, where virtually all patients receive treatment, such distinctions are more difficult.

Many groups have studied prognostic factors in patients presenting with metastatic breast carcinoma. Patients with bone metastases make up the largest single group of patients presenting with metastatic disease. As a consequence, prognostic factors related to this specific group of patients are worthy of more detailed study.

Additional sites of metastatic disease at presentation with bone metastases dramatically worsened survival, with those patients presenting with lung and liver metastases doing significantly worse than those patients presenting with bone metastases alone. Others have also confirmed the importance of site of initial metastases in determining prognosis (Blanco *et al*, 1990; Robertson *et al*, 1992; Coleman *et al*, 1998; Solomayer *et al*, 2000).

Factors that indicate a less aggressive tumour phenotype were associated with the development of bone metastases. Low-grade and ER-positive tumours were more likely to be associated with the development of bone metastases. Others have also found similar relationships (Koenders *et al*, 1991; Coleman *et al*, 1998; Solomayer *et al*, 2000). The same factors were also found to influence survival in patients presenting with bone metastases. Those with low-grade and ER-positive tumours showed significantly prolonged survival compared with patients with high-grade or ER-negative tumours. A shorter metastasis-free interval also had a negative impact on survival in patients with bone metastases. This confirms previous work recognising the prognostic importance of the metastasis-free interval (Solomayer *et al*, 2000). A short metastasis-free interval also tends to suggest a more aggressive, rapidly growing tumour.

The multivariate analysis showed ER status rather than histological grade as an independent prognostic factor for survival in patients presenting with bone metastases. The close relationship between ER status and grade is well recognised with ER activity occurring more frequently in well-differentiated tumours (Williams *et al*, 1987). The reason ER status correlates better with survival is in large part due to the fact that ER status predicts for response to endocrine therapy. The ER is involved in the actual pathway inhibited by endocrine therapy, whereas histological grade is a composite measure of three factors (tubule formation, nuclear pleomorphism and frequency of mitoses) which are themselves not based on a single cellular process. It is perhaps not surprising that ER status is better than histological grade at predicting survival.

One rather curious association was that patients with bone metastases were more likely to have had lymph node metastases at the time of diagnosis of their primary tumour. This may just be a chance finding, but may also be explained by the close relationship between ER status and histological grade. Bone metastases are more likely to occur in ER-positive tumours. There is a strong correlation between ER positivity and well-differentiated tumours (Williams *et al*, 1987). Well-differentiated tumours are unlikely to metastasise unless lymph node positive, unlike poorly differentiated lymph node negative tumours which will still often metastasise. This may be a possible explanation for the association between lymph node metastases and bone metastases.

It is well established that for primary breast cancer lymph node stage, histological grade and to a lesser extent tumour size are powerful prognostic features. As has also been previously demonstrated (Williams *et al*, 1986; Robertson *et al*, 1992), once a patient develops metastatic disease then neither the lymph node status nor the size of the primary tumour is relevant as predictors of survival.

It is well known that the metastatic patterns of lobular and ductal carcinomas are different, with gastrointestinal and peritoneal metastases being more common in lobular carcinomas (Dixon *et al*, 1991). There has been debate in the literature as to whether tumour type has any bearing on the development of bone metastases. Tubular mixed primary tumours were more likely to develop bone metastases compared to Ductal NST tumours. This finding helps to support the argument that less-aggressive tumour phenotypes are more likely to be associated with the development of bone metastases. In common with several previous studies, we found no evidence that patients with lobular carcinoma were more likely to develop bone metastases compared to patients with ductal carcinomas (Dixon *et al*, 1991; Fondrinier *et al*, 1997). Others have suggested that lobular carcinomas have a tendency to metastasise to bone (Borst *et al*, 1993; Sastre-Garau *et al*, 1996).

Patient age does not appear to be a factor in the development of bone metastases, with bone metastases distributed fairly evenly across the age ranges. Others have also confirmed the lack of an age-related trend in the development of bone metastases (De la Monte *et al*, 1988). It has previously been observed that older women are more likely to have metastatic disease that remains confined to bone (Coleman *et al*, 1998). Along with others we have also found that survival for women with bone metastases was significantly worse over the age of 70 years (Coleman *et al*, 1998). It is difficult to know whether this reflects differences in treatment, or the increased likelihood of dying from other conditions. We have seen a similar trend for worsening survival in our elderly patients with liver metastases, which seems in part explained by differences in treatment, with only 6.4% receiving chemotherapy compared to 56% of patients under 70 years (Wyld *et al*, 2002). Death from comorbid conditions has been put forward as an argument for the apparent poor survival in individuals over 70 years of age (Yanick *et al*, 2001). There may even be a link in some patients. For example, older patients have a higher incidence of osteoporosis, which in the presence of bone metastases may result in an increased incidence or earlier fracture rate. Fractures of long bones are known to carry significant mortality in the elderly.

The radiographic appearance of the bone metastases was not related to grade, ER status or type of primary tumour, nor did it have any bearing on patient survival. The only radiographic feature that did impact on patient survival was the number of hot spots on bone scan. Patients with a solitary hot spot on bone scan had significantly improved survival compared to patients with two or more hot spots. It has previously been reported that patients who have bone recurrence at initially only one or two sites on bone scan experience a survival advantage over those with more extensive skeletal metastases (Jacobson *et al*, 2001). Care needs to be taken when interpreting this finding, as a possible explanation for the apparent better outcome may be that a solitary hot spot on a bone scan has been incorrectly classified as malignant. Another explanation may relate to the reason for doing the bone scan, for instance, a patient with an asymptomatic tumour marker rise; in such a case, the apparent improved survival may simply be ‘lead-time’ bias.

The usefulness of blood tumour markers in the diagnosis and monitoring of response to treatment in patients with metastatic disease is well established (Breast Speciality Group of BASO, 1999; Robertson *et al*, 1999; Cheung *et al*, 2000, 2001). Much less has been written about the prognostic value of tumour markers. Correlations between raised preoperative levels of CA15.3 and CEA with poorer outcome have been reported (Molina *et al*, 1998; Shering *et al*, 1998; Ebeling *et al*, 1999; McLaughlin *et al*, 2000; Canizares *et al*, 2001). To our knowledge, the prognostic significance of an elevated CA15.3 and CEA in patients presenting with bone metastases has not been previously described. Several authors have commented on the correlation between overall tumour burden and CA15.3 levels (O'Hanlon *et al*, 1995; Tampellini *et al*, 2001). It is likely that elevated tumour markers reflect tumour burden in patients presenting with bone metastases.

Analysis of the subgroup with bone as their only site of metastatic disease revealed that patients with ER-negative tumour, elevated CA 15.3 level at presentation and a short metastasis-free interval experienced significantly reduced survival. This may have important implications for treatment, as patients with bone-only disease are traditionally treated with endocrine therapies alone. Patients with bone-only metastatic disease with ER-negative tumours, elevated CA15.3 levels at presentation and a short metastasis-free interval may benefit from more aggressive treatments, possibly with chemotherapy being considered as a first-line agent.

Prognostic factors are important for patient counselling and advising treatment options. This study has revealed several potential independent prognostic factors in patients presenting with bone metastases. These are ER status, metastasis-free interval, additional sites of metastases other than bone and elevated tumour marker levels. The factors that suggest a more aggressive tumour phenotype, such as negative ER status and short metastasis-free interval, are associated with a poorer outcome. Factors that are associated with a greater tumour burden also predict for reduced survival, these are raised tumour markers and the presence of extraosseus disease. Factors that suggest hormone sensitivity (a positive ER status) predict for longer survival. Awareness of the prognostic implications of these factors may aid the selection of the most appropriate treatment options in patients presenting with bone metastases.
